# Cardiac Autonomic Modulation and Anti-Thyroid Peroxidase (TPO) Antibodies in Subclinical Hypothyroidism: Does a Correlation Exist?

**DOI:** 10.7759/cureus.18844

**Published:** 2021-10-17

**Authors:** Manisha Mavai, Bharti Bhandari, Anish Singhal, Sandeep K Mathur

**Affiliations:** 1 Physiology, Government Medical College, Bharatpur, IND; 2 Physiology, Government Institute of Medical Sciences, Greater Noida, IND; 3 Physiology, All India Institute of Medical Sciences, Bibinagar, IND; 4 Endocrinology, Sawai Man Singh Medical College, Jaipur, IND

**Keywords:** anti tpo antibodies, thyroid dysfunction, sympathovagal imbalance, subclinical hypothyroidism, heart rate variability, cardiac autonomic activity

## Abstract

Background: Heart rate variability (HRV) reflects the balance between the sympathetic and parasympathetic divisions of the autonomic nervous system. Anti-thyroid antibodies like anti-TPO and anti-Thyroglobulin have long been associated with thyroid dysfunction and abnormal thyroid profile testing. Subclinical hypothyroidism (SCHypo) is characterized by elevated thyroid-stimulating hormone (TSH) with normal thyroid hormones. We hypothesize that autonomic function may be deranged in anti-TPO positive sub-clinical hypothyroid cases, even before the onset of overt hypothyroidism.

Objectives: To investigate the association between anti-Thyroid Peroxidase antibodies (anti-TPOAb) positive SCHypo and sympathovagal imbalance (SVI), if any.

Methodology: The study was conducted on the age and body mass index (BMI) matched subclinical hypothyroid patients (n=52) and healthy controls (n=20). The cardiac autonomic activity was assessed by short-term HRV in the time (SDNN, RMSSD, pNN50) and frequency domains (LFms^2^, HFms^2^, LFnu, HFnu, TP, and LF/HF ratio). Nonlinear geometric measures (SD1, SD2, SD1/SD2, TINN, HRV triangular index) were also evaluated. Biochemical evaluation of serum thyroid profile and anti-TPOAb was done in all the subjects.

Results: Decreased HRV was observed in the anti-TPOAb positive group when compared to the antibody-negative and control groups. Significant positive correlation of anti-TPOAb with TSH, LFnu, LF/HF and negative correlation with SDNN, RMSSD, pNN50, SD1, SD1/SD2, HFnu, and TP of HRV was observed.

Conclusion: Anti-TPOAb positive SCHypo group exhibited modifications in HRV characterized by decreased parasympathetic modulation, as compared to controls. The findings were also suggestive of increased risk of autonomic dysfunction in TPOAb-positive patients, as compared to antibody negative. An increase in anti-TPO antibodies was significantly correlated with TSH and SVI in SCHypo patients.

## Introduction

Subclinical Hypothyroidism (SCHypo) is defined as a clinical entity in which the serum-free thyroxine (fT4) level remains within the reference range with increased serum thyroid-stimulating hormone (TSH) level [1]. Autoimmune thyroid diseases include hyperthyroid Grave’s disease, hypothyroid autoimmune thyroiditis, and subtle sub­clinical thyroid dysfunctions [2]. Thyroid autoimmunity is characterized by easily detectable production of thyroid autoantibodies, to thyroglobulin (TG) and thyroid peroxidase (TPO) [3]. TPOAbs are the circulating hallmark of autoimmune thyroid disease and are present in a majority of cases [4]. 

The thyroid gland and the Autonomic Nervous System are closely linked by their effects on the cardiovascular system [5,6]. The ANS controls the heart through a complex mechanism of interactions between sympathetic and parasympathetic divisions, producing fluctuations in heartbeat intervals. The greater the fluctuations in the beat-to-beat interval, the better the cardiovascular system functions to adapt and respond to internal and external stimuli [7]. Heart rate variability (HRV) is an easy, non-invasive, sensitive, and widely applied method for cardiac autonomic assessment. Lowered HRV is associated with an increased risk of mortality, and HRV has been proposed as a marker for cardiovascular disease [8]. 

Autonomic disturbances are common in patients with overt thyroid diseases [6,9]. Even in subclinical conditions, an increased number of cardiovascular risk factors and altered autonomic activity have been observed [10,11]. It has been suggested that clinical impairments in SCHypo may precede cardiac dysfunctions [10,12]. With the above background, this study was planned to assess the cardiac autonomic functions by HRV parameters in anti-TPOAb positive and anti-TPOAb negative subclinical hypothyroid subjects and to assess the association between the presence of anti-TPOAb in SCHypo and sympathovagal imbalance (SVI).

## Materials and methods

The study commenced after obtaining formal approval from Institutional Ethics Committee. Informed written consent was obtained from all the subjects included in the study. Age and body mass index (BMI) matched subclinical hypothyroid patients and healthy controls, 20-50 years of age, never previously treated for any endocrine disease, and ready to participate were included in the study. The sample size was based on convenient sampling. All patients fulfilling the inclusion criteria and who visited the OPD during the 10-months study period were included in the study. The participants were stratified into anti-TPOAb positive (n= 35), anti-TPOAb negative (n= 17) and control (n=20) groups. SCHypo was defined as the patients with a TSH level of 4.5-10 mU/ml and the normal individuals with a history of complaints of any cardiac, hepatic, or renal dysfunction, HIV/Immunodeficiency disorders, neurological disease, any other systemic disease that may affect autonomic activity, i.e., diabetes, hypertension, while those taking any drug which affects autonomic activity were excluded. Serum levels of fT3, fT4, TSH, anti-TPOAb levels were measured by using the chemiluminescent immunoassay method (IMMULITE 2000 Systems Analyzer). The serum fT3, fT4, TSH levels for the euthyroid state were between 1.8-4.2 pg/mL, 0.89-1.76 ng/dL and 0.4-4.0 μIU/mL, respectively. The normal level of anti-TPOAb was <35 IU/mL. 

**Figure 1:**
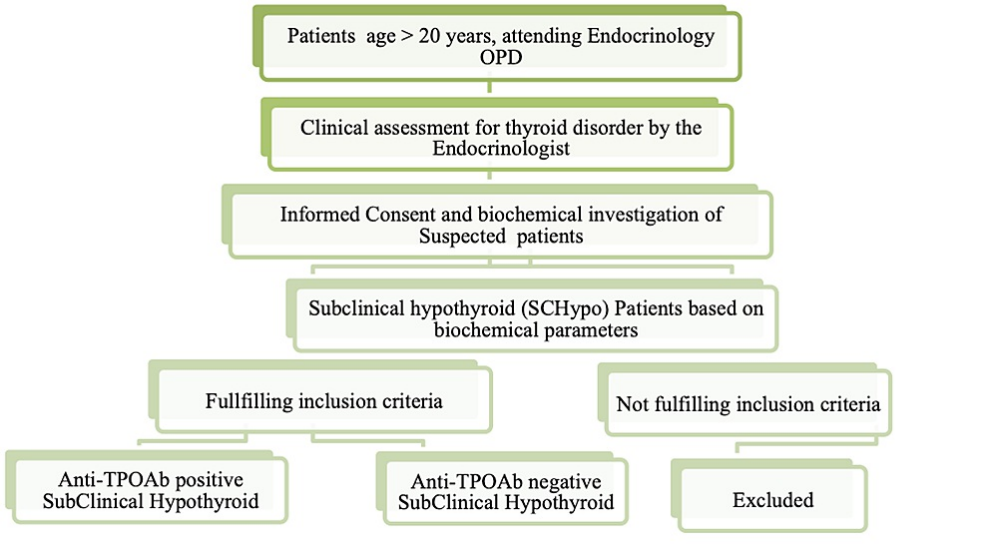
Recruitment and screening planning

Based on the above-mentioned criteria, the following recruitment/screening plan was charted (Figure 1):

Based on the findings of the screening, the patients were grouped as anti-TPOAb positive subclinical hypothyroid (n=35), anti-TPOAb negative subclinical hypothyroid (n=17), and age and BMI matched healthy subjects (n=20). The healthy subjects were asymptomatic with normal clinical examination and biochemical tests and not on any medication, randomly selected from the general population. Confirmation of clinical diagnosis was done by biochemical evaluation of FT3, FT4, TSH, anti-TPO antibodies.

Heart rate variability assessment was done by recording five minutes ECG using an RMS electrocardiograph (DECG 1/ 63041/ ADBXB, Recorders & Medicare Systems Pvt. Ltd., India). The analogue signals were converted to digital signals by National Instruments Software NI-DAQ Version 8.0 (National Instruments Corp., India). The analysis was performed using linear methods, which were analyzed in the time domain, frequency domain, and by the geometric measures. Linear analysis of HRV for time domain (SDNN, RMSSD, pNN50%), and frequency domain (total power, LF (ms^2^), HF (ms^2^), HF in the normalized unit (nu), LF (nu), LF/HF ratio) parameters was performed by HRV analysis software version 1.1 (Bio-signal Analysis group, Kuopio, Finland).

The time-domain analysis included the squared root of the mean of the sum of the squares of successive normal R-R interval differences (RMSSD) and the percentage number of pairs of adjacent normal R-R intervals differing by more than 50 ms in the entire recording (pNN50), and these were used as indexes of parasympathetic activity. We also computed the standard deviation of all normal R-R intervals (SDNN).

In the frequency domain, parameters included total power (TP) of HRV spectrum, the power in the high (HF) and the low frequency (LF) bands in absolute units (ms2), HFnu, LFnu, and the LF-HF ratio [8].

In the geometric measures, the Poincaré plot was evaluated quantitatively through the computation of the SD indexes of the plot. Poincaré indices, SD1 (standard deviation of the instantaneous beat-to-beat variability), SD2 (standard deviation of the long-term continuous RR intervals); SD1/SD2 ratio; and TINN (triangular interpolation of NN intervals); HRV index were analyzed [9].

Experimental protocol

(i) For HRV, the participants underwent five minutes of rest in the supine position. The recording was done in a quiet and comfortable room, and the temperature was maintained at 24-28°C. The subject was instructed to close the eyes and to avoid talking, moving hands, legs, and body, coughing during the test, and sleeping.

(ii) The subjects were instructed to avoid food intake at least two hours before the procedure, abstain from coffee, nicotine, or alcohol 24 hours before testing, and wear loose and comfortable clothing. They were asked to report at 10.00 am. Their age, height, and body weight were recorded. Body Mass Index (BMI) was calculated by weight (kg) divided by the square of height (meter) (Quetelet’s Index). 

(iii) All standard limb leads were applied, and the lead with an upright R wave was selected for recording. The ECG signals were continuously amplified, digitized, and stored in the computer for offline analysis. R wave and RR interval detection were done by the software. Abnormal beats and areas of artifact were automatically identified and excluded from the recording.

Statistical analysis

Statistical analysis was performed using GraphPad Prism version 5 (GraphPad Software Inc., San Diego, California). Descriptive characteristics are presented as means and standard deviations (mean±SD). Kolmogorov-Smirnov test was done to assess the normal distribution of variables. For parametric data, one-way ANOVA (analysis of variance) followed by post-hoc Bonferroni test and for non-parametric data, Kruskal-Wallis one-way ANOVA with post hoc Dunn’s multiple comparisons tested the level of significance among the groups. Spearman Rank correlation tests were done to study associations between various variables. For all the analyses, statistical significance was defined at the level of p <0.05.

## Results

The study population consisted of 52 sub-clinical hypothyroid patients and 20 controls. Based on the presence of thy­roid autoantibody (anti-TPOAb) status, the sub-clinical hypothyroid subjects were divided into two groups: anti-TPOAb positive group (n=35) and anti-TPOAb negative group (n=17). The general and other characteristics of the groups are given in Table 1. TSH was statistically higher in the test group than the control group (p<0.001) whereas, age, BMI, free T3, free T4 were comparable. Anti-TPO antibody was found to be statistically higher in the antibody-positive group as compared to antibody negative and controls (p<0.001).

**Table 1:** Age, BMI, thyroid profile and anti-TPOAb parameters of control, anti-TPOAb negative and anti-TPOAb positive subclinical hypothyroid subjects Data is presented as Mean±SD. P<0.05 was considered statistically significant. Intergroup comparison was done using one-way ANOVA followed by a post hoc Bonferroni multiple comparison test (age, BMI), and Kruskal–Wallis followed by Dunns multiple comparison test (Thyroid profile and Anti-TPOAb). ns: not significant. *Control vs anti-TPOAb negative. † Control vs anti-TPOAb positive. ‡ anti-TPOAb positive vs anti-TPOAb negative. BMI: body mass index; TSH: thyroid-stimulating hormone; anti-TPOAb: anti-thyroid peroxidase antibodies; SCHypo: subclinical hypothyroidism.

Parameters (Mean ± SD)	Control (n=20)	Anti-TPOAb negative SCHypo (n=17)	Anti-TPOAb positive SCHypo (n=35)	p-value	Intergroup comparison p value
Age (yrs.)	34.44±10.0	32.12±8.49	34.05±7.25	>0.05	ns*†‡
BMI (kg/m^2^)	21.3±1.11	22.33±2.62	22.34±1.68	>0.05	ns*†‡
FreeT3 (pg/mL)	2.59±0.47	2.89±0.25	2.78±0.72	>0.05	ns*†‡
Free T4 (ng/dL)	1.21±0.19	1.12±0.22	1.10±0.31	>0.05	ns*†‡
TSH (uIU/mL)	2.41±1.15	6.47±2.20	6.68±3.12	<0.001	<0.001*†, ns‡
Anti-TPOAb (IU/mL)	22.96±4.6	25.39±8.12	597.4±454.8	<0.001	ns*, <0.001†‡

HRV indices analyzed in the time, frequency domain, and nonlinear geometric measures and their intra- and inter-group comparison are given in Table 2. TPOAb-positive patients had significantly lower SDNN, pNN50, and TINN values compared to controls, though RMSSD and HRV triangular index were found to be nonsignificant. Furthermore, in the TPOAb-positive SCHypo, HFnu and SD1 were significantly lower, whereas SD2 was significantly higher than controls. TP was also significantly decreased in the TPOAb-positive group than controls. The LF/HF, SD1/SD2 exhibited a significant change in anti-TPOAb positive patients compared to controls.

**Table 2:** Time and frequency domain measures of HRV in control, anti-TPOAb negative and anti-TPOAb positive subclinical hypothyroid subjects Data is presented as Mean±SD. P<0.05 was considered statistically significant. SDNN: standard deviation of normal to normal interval, RMSSD: the square root of the mean of squares of the differences between adjacent NN intervals, pNN50: the proportion derived by dividing NN50 by the total number of NN intervals, LF: low-frequency power (ms^2^), HF: high-frequency power (ms^2^), LFnu: normalized low-frequency power, HFnu: normalized high-frequency power, LF/HF: ratio of LF to HF, TP: total power, SD1/ SD2: ratio of SD1 to SD2, TINN: triangular interpolation of NN interval; anti-TPOAb: anti-thyroid peroxidase antibodies; SCHypo: subclinical hypothyroidism. Intergroup comparison was done using Kruskal–Wallis followed by Dunns multiple comparison test. ns: not significant. *Control vs anti-TPOAb negative. † Control vs anti-TPOAb positive. ‡ anti-TPOAb positive vs anti-TPOAb negative.

Parameter (Mean ± SD)	Control (n=20)	Anti-TPOAb negative SCHypo (n=17)	Anti-TPOAb positive SCHypo (n=35)	p-value	Intergroup comparison p values
SDNN (ms)	53.43±14.22	48.02±9.42	42.2±6.43	<0.01	ns*‡, <0.01†
RMSSD (ms)	54.03±20.35	50.12±9.63	43.15±17.79	>0.05	ns*†‡
pNN50 (ms)	25.63±7.79	20.52±18.81	11.17±6.4	<0.001	ns*‡, <0.001†
LF (ms^2^)	994.8±632.3	865.2±460.3	618.5±310.7	>0.05	ns*‡†
HF (ms^2^)	1298±681.7	1058±801.5	922.1±316.7	>0.05	ns*†‡
LFnu	40.89±9.41	45.61±8.55	47.25±10.72	>0.05	ns*†‡
HFnu	61.33±14.81	55.68±12.0	50.20±13.36	<0.05	ns*‡, <0.05†
LF/HF	0.75±0.36	1.01±0.40	1.2±0.52	<0.01	ns*‡, <0.01†
TP	3482±1183	2724±768.1	2178±956.9	<0.001	ns*‡, <0.001†
SD1 (ms)	36.82 ± 16.68	25.47 ± 14.59	17.71 ± 11.24	<0.0001	ns*‡, <0.0001†
SD2 (ms)	40.51 ± 20.85	46.70 ± 20.11	59.19 ± 26.78	<0.05	ns*‡, <0.05†
SD1/SD2	1.18 ± 0.79	0.56 ± 0.33	0.47 ± 0.55	<0.0001	ns*‡, <0.0001†
HRV Index	0.40 ± 0.26	0.31 ± 0.24	0.27 ± 0.21	>0.05	ns*‡†
TINN	338.6 ± 256.6	222.4 ± 102.7	161.6 ± 71.56	<0.01	ns*‡, <0.01†

Table 3 presents the correlation of TPO with TSH and HRV indices. A positive correlation of anti-TPOAb with TSH, LFnu, and LF/HF was observed, and negative with HFnu, TP, SDNN, RMSSD, and pNN50. Other HRV parameters also correlated with anti-TPOAb.

**Table 3:** Correlation of anti-TPOAb with TSH & HRV parameters P<0.05 was considered statistically significant. Spearman rank correlation test was used. ‘*’ denotes a significant correlation. Minus R-values depict negative correlation and plus sign shows positive correlation. TSH: thyroid-stimulating hormone, LF (ms^2^): low-frequency power (ms2), LFnu: normalized low-frequency power, HF (ms^2^): high-frequency power (ms2), HFnu: normalized high-frequency power, TP: total power, LF/HF: ratio of LF to HF, SDNN: standard deviation of normal to normal interval, RMSSD: the square root of the mean of squares of the differences between adjacent NN intervals, pNN50: the percentage number of pairs of adjacent normal R-R intervals differing by more than 50 ms in the entire recording, SD1: (standard deviation of the instantaneous beat-to-beat variability), SD2: (standard deviation of the long-term continuous RR intervals, SD1/ SD2: ratio of SD1 to SD2, HRV Index: heart rate variability index, TINN: triangular interpolation of NN interval.

Parameters	R	p value
TSH	0.963	<0.0001*
LF (ms^2^)	-0.248	0.169
LFnu	0.635	<0.0001*
HF (ms^2^)	-0.146	0.425
HFnu	-0.606	0.002*
TP	-0.639	<0.0001*
LF/HF	0.757	<0.0001*
SDNN	-0.999	<0.0001*
RMSSD	-0.999	<0.0001*
pNN50	-0.999	<0.0001*
SD1	-0.786	<0.0001*
SD2	0.831	<0.0001*
SD1/SD2	-0.999	<0.0001*
HRV Index	-0.178	0.3462
TINN	-0.356	0.0535

## Discussion

The study compared the autonomic activity in TPOAb-positive and TPOAb-negative groups. The anti-TPO antibody-positive group demonstrated greater alterations in autonomic modulation, primarily characterized by diminished parasympathetic activity. A literature search could not retrieve any study on the anti-TPOAb status of SCHypo patients and its correlation with HRV. However, available research findings have shown significantly higher sympathetic and lower parasympathetic activity at baseline conditions in SCHypo [10-12].

No significant difference was observed in TSH levels between anti-TPOAb positive and anti-TPOAb negative patients, though there was a significant difference in the TSH levels between the control and SCHypo groups. Contrary to this finding, Brown J (2016) evidenced significantly higher serum TSH in antibody-positive than in antibody-negative individuals [13].

In the current study, a significant increase in the LF-HF ratio in the anti-TPOAb positive SCHypo patients was recorded in comparison to the control group, though no significant difference was observed between control and anti-TPOAb negative patients. The LH-HF ratio is a sensitive marker of SVI [8]; hence, the increase in the LF-HF ratio represents considerable SVI in anti-TPOAb positive SCHypo. In the Poincaré indices (SD1 and SD2) of HRV, the width of SD1 reflects the parasympathetic modulation and the SD2 length reflects the sympathetic activity [14]. In our study, the SD1 value was profoundly decreased, and SD2 was increased in the antibody-positive SCHypo group than in controls, which can be used as a sensitive indicator of sympathovagal changes. Akin to the HF-LF ratio, the SD1-SD2 ratio also reflects the sympathovagal balance. The significant decrease in SD1/SD2 in the anti-TPOAb positive group further confirms SVI in TPOAb positive SCHypo.

In anti-TPOAb positive SCHypo patients, a significant decrease in HFnu indicates decreased parasympathetic activity in these patients, as HFnu is an index of cardiac vagal drive [8,14]. This was supported by a significant reduction in TP of HRV in antibody-positive SCHypo patients compared with controls, like TP, in general, indicates the magnitude of vagal modulation of cardiac function [8,15].

The time-domain indices of HRV: SDNN, RMSSD, and pNN50 were significantly decreased in anti-TPOAb positive SCHypo patients than in controls, further confirming the decreased vagal tone in these patients. The reduction in parasympathetic activity in the TPO-positive group compared to healthy individuals can be further verified by the decrease in the SD1 indices. Hence, the anti-TPO positive SCHypo group showed lower parasympathetic modulation than the control group. A decreased vagal activity is related to an increased risk for all-cause morbidity and mortality, and for the development of numerous other risk factors [16].

In this study, no significant differences in cardiac vagal activity were observed in the two SCHypo groups though the TPOAb-positive group showed lower values in magnitude for parasympathetic indices in comparison to the TPOAb-negative group. This points towards a likelihood of sympathovagal imbalance (SVI) in the anti-TPOAb positive SCHypo group. Thus, from the present study, it may be presumed that SVI in the anti-TPO positive SCHypo could be due to diminished vagal activity presuming that SVI in anti-TPO positive SCHypo could be due to diminished vagal activity. In a previous study, reduced time domain indices and lower HF values in frequency domain analysis in SCHypo patients were found in comparison to controls [11].

In our study, anti-TPOAb were found to correlate positively with TSH and HRV indices. To the best of our knowledge, no study has assessed the correlation of anti-TPOAb levels in SCHypo patients with HRV indexes (linear as well as nonlinear). It has been proved that anti-TPOAb negative autoimmune thyroiditis has a milder course, while the presence of anti-TPOAb is associated with an increased risk of overt hy­pothyroidism [17,18]. It has been postulated that the presence of antibodies is linked to an increased risk of atherosclerotic disease and myocardial infarction [19]. Furthermore, decreased parasympathetic activity has also been proposed as an important risk factor for cardiovascular disease and mortality [6]. Hence, anti-TPOAb positive SCHypo patients are more prone to cardiac risk than anti-TPOAb negative SCHypo patients; this has been proved further by this study.

The study is limited by the fact that we could not assess the cardiac autonomic reactivity by Conventional Autonomic Function Tests. Also, we have not studied the cardiac dysfunctions by echocardiography and their possible correlation with anti-TPOAb levels in subclinical hypothyroid subjects. Future studies should be planned to confirm our findings in a large sample size.

## Conclusions

Based on our study findings, it can be concluded that patients of subclinical hypothyroidism, who are anti-thyroid peroxidase antibody positive, have deranged heart rate variability (HRV) and sympathovagal imbalance (SVI), which makes them more prone to cardiovascular morbidity and mortality. The deranged HRV and SVI make them more prone to develop cardiovascular disease and morbidity. It is recommended that anti-TPOAb-positive SCHypo patients should be assessed early for cardiovascular involvement to prevent further progression of cardiac disease and to prevent cardiovascular morbidity and mortality.
